# Disseminated Skeletal Tuberculosis Presenting as Multifocal Axial and Peripheral Joint Disease: A Diagnostic Challenge

**DOI:** 10.7759/cureus.105351

**Published:** 2026-03-16

**Authors:** Tarikul Islam, Ahmed S Alsherbeeny, Latif Rahman, Tanmoy K Saha, Mehedy Hasan

**Affiliations:** 1 Internal Medicine, Sher-e-Bangla Medical College Hospital, Barishal, BGD; 2 Acute Medicine, University Hospitals of Leicester, Leicester, GBR; 3 Internal Medicine, Dhaka Medical College and Hospital, Dhaka, BGD

**Keywords:** diagnostic challenge, disseminated tuberculosis, infectious arthritis, spinal tuberculosis, tuberculous arthritis

## Abstract

Disseminated skeletal tuberculosis (TB) is an uncommon manifestation of extrapulmonary TB and often presents with non-specific musculoskeletal symptoms, contributing to diagnostic delay and an increased risk of irreversible structural damage. We report a 35-year-old male with diabetes mellitus who presented with two months of progressive nocturnal low back pain with functional limitation, followed by progressive painful swelling and restricted movement of the left elbow, and subsequently developed high-grade fever, weight loss, and constitutional symptoms. The coexistence of axial and peripheral skeletal involvement is uncommon and contributed to the diagnostic challenge in this case. Magnetic resonance imaging demonstrated vertebral destruction centred at the L4 level with pre- and paravertebral soft tissue involvement consistent with spinal TB, sacroiliitis with an associated psoas collection suggestive of abscess formation, and erosive changes of the left elbow with extensive periarticular soft tissue involvement mimicking septic arthritis. CT-guided tissue sampling revealed granulomatous inflammation supporting a diagnosis of disseminated skeletal TB in the appropriate clinical and radiological context. The patient was treated with prolonged first-line anti-tuberculous therapy with early clinical and biochemical improvement. This case highlights the diagnostic challenge posed by multifocal skeletal TB involving both axial and peripheral joints and underscores the importance of early consideration of TB, comprehensive imaging evaluation, and timely tissue diagnosis to enable appropriate treatment and prevent long-term morbidity.

## Introduction

Tuberculosis (TB) remains a major global public health concern, with an estimated 10 million new cases annually and extrapulmonary TB accounting for approximately 15-20% of reported disease worldwide, particularly in endemic regions and in patients with chronic comorbidities [1,2]. Bangladesh remains among high TB-burden countries, and extrapulmonary TB continues to contribute substantially to morbidity in the region [1]. Despite improvements in diagnostic strategies and treatment outcomes, extrapulmonary TB continues to present significant clinical challenges due to its heterogeneous manifestations and frequent delays in definitive diagnosis [2,3].

Musculoskeletal TB is an uncommon but clinically important form of extrapulmonary TB, representing approximately 1-3% of all TB cases and 10-15% of extrapulmonary disease [3,4]. The spine is the most frequently involved skeletal site, while large weight-bearing joints are affected less commonly; involvement of peripheral joints and the sacroiliac joint remains rare and is often under-recognised [4,5]. The disease typically follows an indolent course, with non-specific symptoms that contribute to delayed diagnosis and an increased risk of irreversible structural damage [5,6]. Multifocal skeletal TB involving both axial and peripheral joints is even more uncommon and may further obscure the diagnosis because it can mimic septic arthritis, inflammatory arthropathies, and malignant disease.

Clinical and radiological features of skeletal TB frequently overlap with those of septic arthritis, inflammatory spondyloarthropathies, and malignancy, leading to frequent misdiagnosis or inappropriate initial management [6,7]. Constitutional symptoms such as fever, weight loss, and night sweats may be absent or develop late, further complicating early recognition [7]. Magnetic resonance imaging (MRI) is the preferred modality for early assessment due to its high sensitivity for bone marrow involvement, soft tissue extension, and abscess formation; however, imaging findings are not pathognomonic and cannot reliably differentiate TB from pyogenic infection without histological or microbiological confirmation [8,9].

Osteoarticular TB is typically paucibacillary, and definitive diagnosis often relies on tissue sampling demonstrating granulomatous inflammation, with or without microbiological confirmation of *Mycobacterium tuberculosis* [9,10]. Disseminated skeletal TB, defined by involvement of multiple osseous or articular sites, is particularly uncommon and represents a major diagnostic challenge, especially when axial and peripheral joints are affected concurrently [7,11]. Delayed recognition may result in spinal instability, neurological compromise, joint destruction, and long-term functional impairment [5,6]. We report a case of disseminated skeletal TB presenting with simultaneous spinal, sacroiliac, and peripheral joint involvement, highlighting key diagnostic pitfalls and emphasising the importance of early consideration of TB, appropriate cross-sectional imaging, and timely tissue diagnosis to guide definitive management.

## Case presentation

A 35-year-old male with a background of diabetes mellitus presented with a two-month history of progressively worsening low back pain. The pain developed insidiously, was more severe at night, and significantly limited mobility. Approximately two weeks after symptom onset, he developed unilateral buttock pain suggestive of sacroiliac joint involvement. Over the following weeks, his clinical course evolved with the onset of progressive pain and swelling of the left elbow associated with restriction of movement, beginning approximately six weeks prior to admission. Fifteen days before presentation, he developed high-grade fever accompanied by constitutional symptoms, including anorexia and unintentional weight loss. There was no reported history of preceding trauma, inflammatory back pain features, or known contact with an individual with active TB. He reported no recent travel, and HIV screening was negative.

A review of drug history did not reveal exposure to immunosuppressive agents prior to presentation. Specifically, there was no use of systemic corticosteroids, biologic therapies, or Janus kinase (JAK) inhibitors (e.g., tofacitinib or baricitinib).

On examination, he appeared unwell and febrile, but remained haemodynamically stable. Lumbar spine movements were markedly restricted due to pain, with tenderness localised to the lumbosacral region and maximal over the sacroiliac joint. Sacroiliac provocation tests were positive. Examination of the left elbow demonstrated visible swelling with overlying healed skin changes, local warmth, tenderness, and a reduced range of motion, particularly in extension. No peripheral lymphadenopathy was identified. Cardiovascular and respiratory examinations were unremarkable, and there were no focal neurological deficits.

Initial laboratory investigations demonstrated systemic inflammation with leukocytosis and markedly elevated inflammatory markers: total leukocyte count of 15.87 K/µL, erythrocyte sedimentation rate (ESR) of 74 mm/hour, and C-reactive protein (CRP) level of 147.48 mg/L.

**Figure 1:**
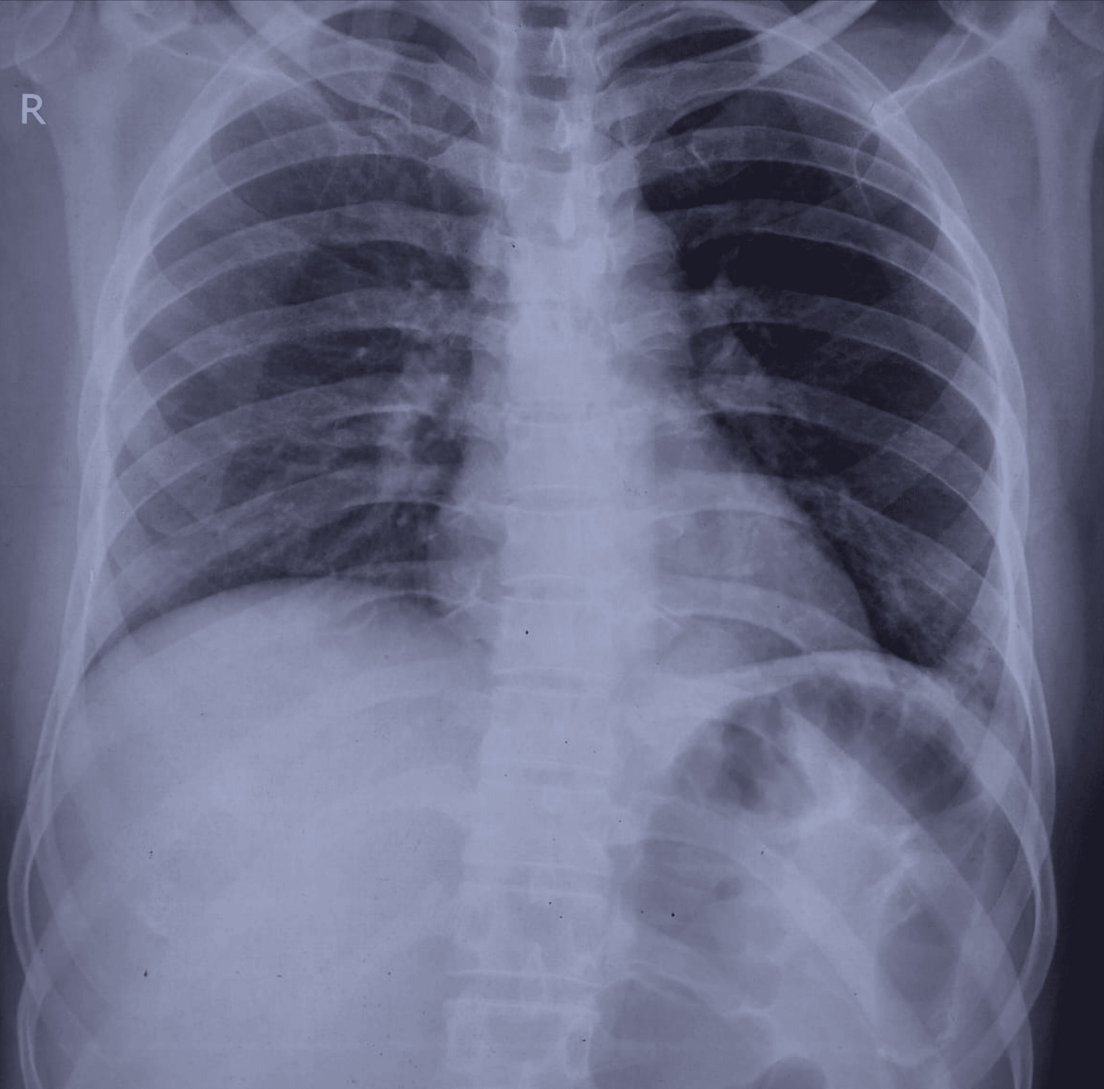
Chest radiograph at presentation. A plain chest radiograph obtained during the initial evaluation demonstrated no clear radiographic evidence of active pulmonary tuberculosis.

**Figure 2:**
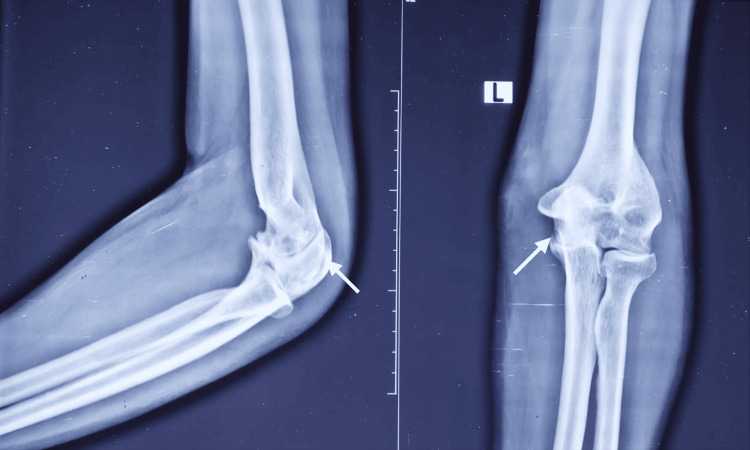
Plain radiographs of the elbow demonstrating erosive articular changes. Radiographs demonstrated periarticular/osseous erosive changes involving the distal humerus and proximal ulna (arrows), supporting an infective or inflammatory arthropathy in the clinical context of progressive painful swelling and restricted movement.

**Figure 3:**
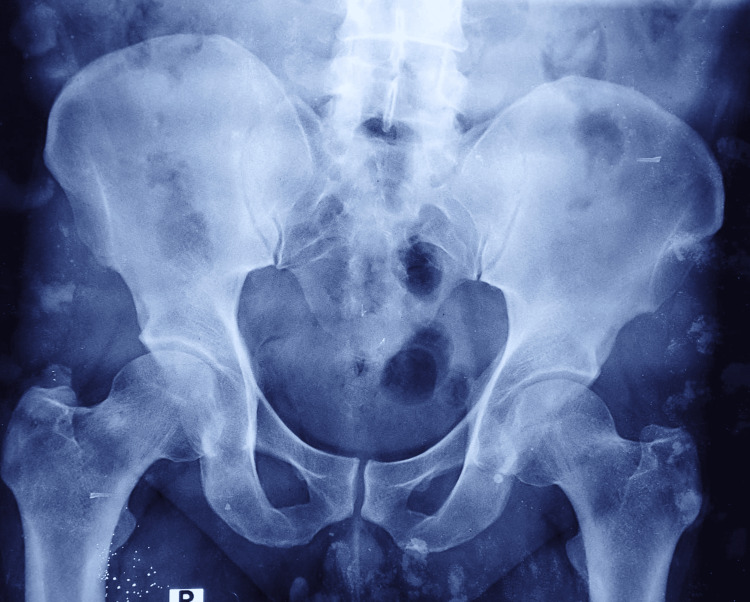
Pelvic radiograph (anteroposterior view). Anteroposterior pelvic radiograph demonstrated no overt destructive changes of the sacroiliac joints. Plain radiography was non-specific, and cross-sectional imaging was required for definitive evaluation.

Initial imaging included a chest radiograph, which did not show clear radiographic evidence of active pulmonary TB (Figure 1). Plain radiographs of the left elbow showed periarticular erosive changes, raising concern for an infective or inflammatory arthropathy in the context of progressive symptoms (Figure 2). A pelvic radiograph was performed as part of the assessment for axial symptoms and sacroiliac joint tenderness (Figure 3). Plain radiographs may remain non-specific in early skeletal TB, and cross-sectional imaging is therefore often required to better characterise the extent of disease.

**Figure 4:**
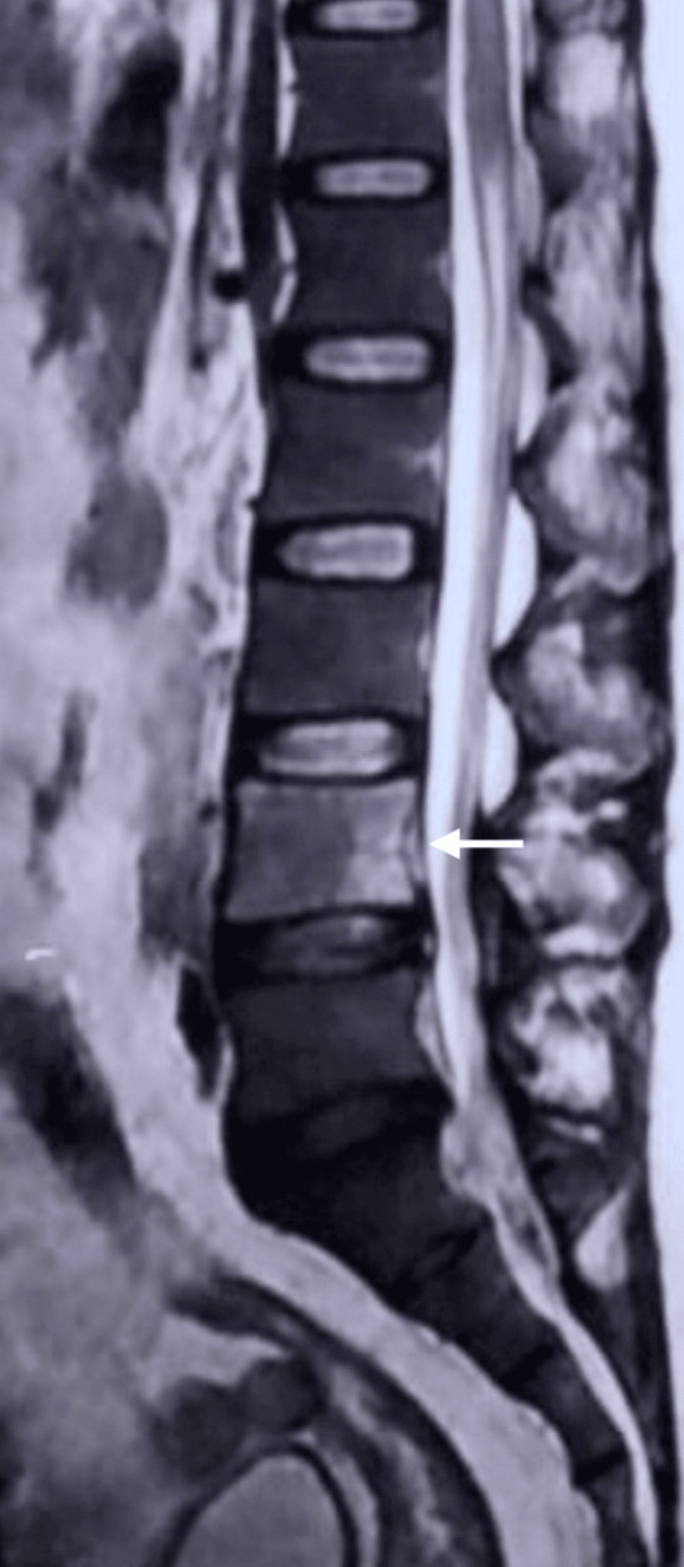
MRI of the lumbosacral spine demonstrating destructive vertebral changes centred at L4. Sagittal MRI image of the lumbosacral spine showed destructive vertebral changes at the L4 level with adjacent inflammatory soft-tissue change. The arrow highlights the L4 vertebral body level corresponding to the site of maximal abnormality, in keeping with spinal infection in the clinical context.

**Figure 5:**
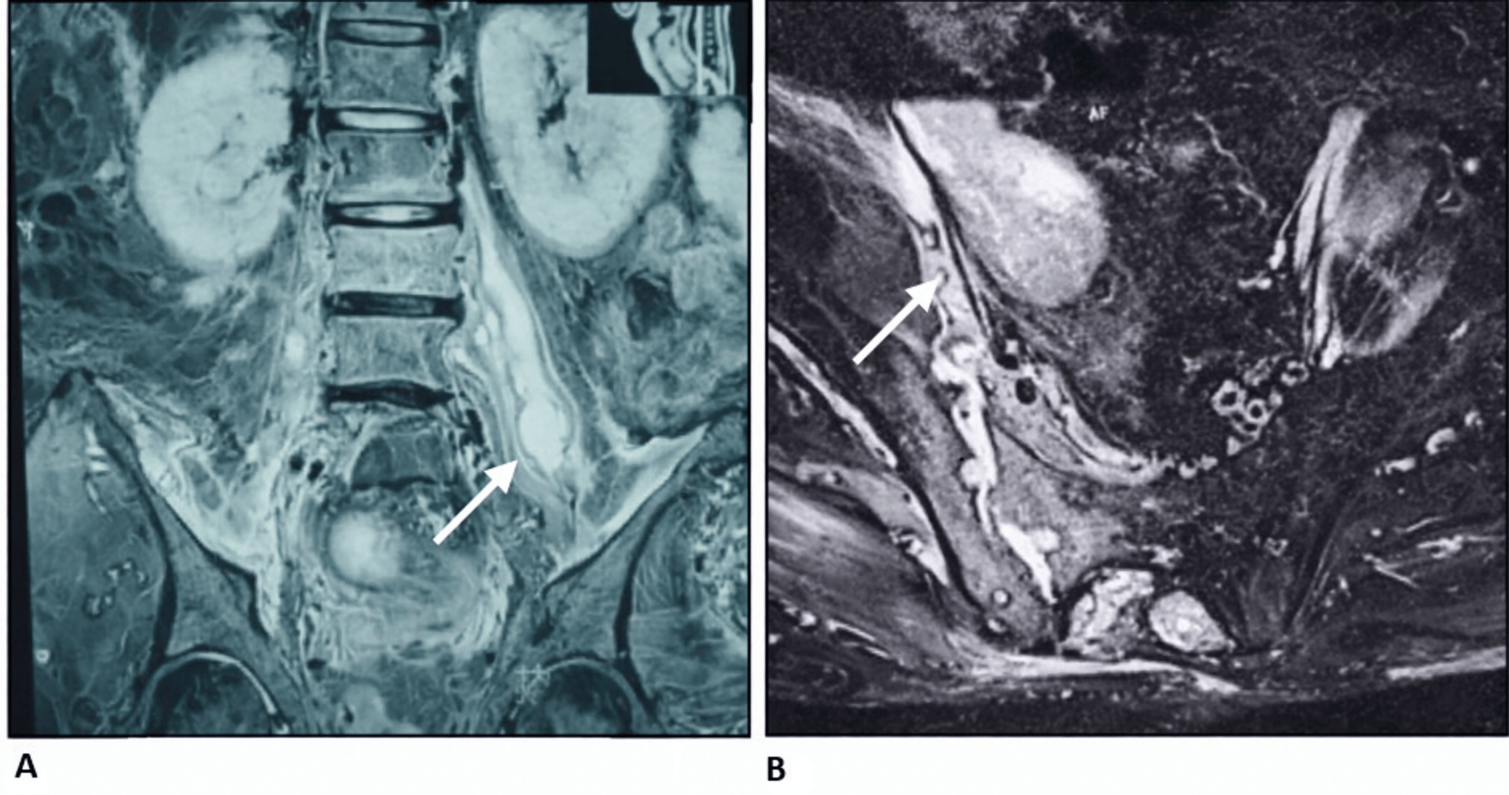
Magnetic resonance imaging of the sacroiliac region demonstrating sacroiliitis with associated psoas collection. (A) Coronal magnetic resonance image demonstrating an associated psoas collection consistent with abscess formation (arrow). (B) Axial magnetic resonance image demonstrating sacroiliac joint involvement with joint space irregularity and adjacent inflammatory change (arrow).

Given the combination of persistent axial skeletal pain, sacroiliac involvement, progressive left elbow arthritis, and systemic features, further cross-sectional imaging was undertaken. MRI of the lumbosacral spine demonstrated destructive vertebral changes centred at the L4 level with associated pre- and para-vertebral soft-tissue involvement, consistent with spinal infection (Figure 4). Although these imaging findings raised a strong suspicion for infectious spondylitis, including a possible tuberculous aetiology, other infectious causes were initially considered during the diagnostic evaluation. MRI of the sacroiliac region demonstrated sacroiliitis with a collection extending along the psoas muscle, consistent with abscess formation (Figure 5).

At this stage, given vertebral involvement and the presence of a psoas-region collection, alternative chronic infective causes were considered. Brucella serology was performed and was negative, supporting an alternative aetiology.

**Figure 6:**
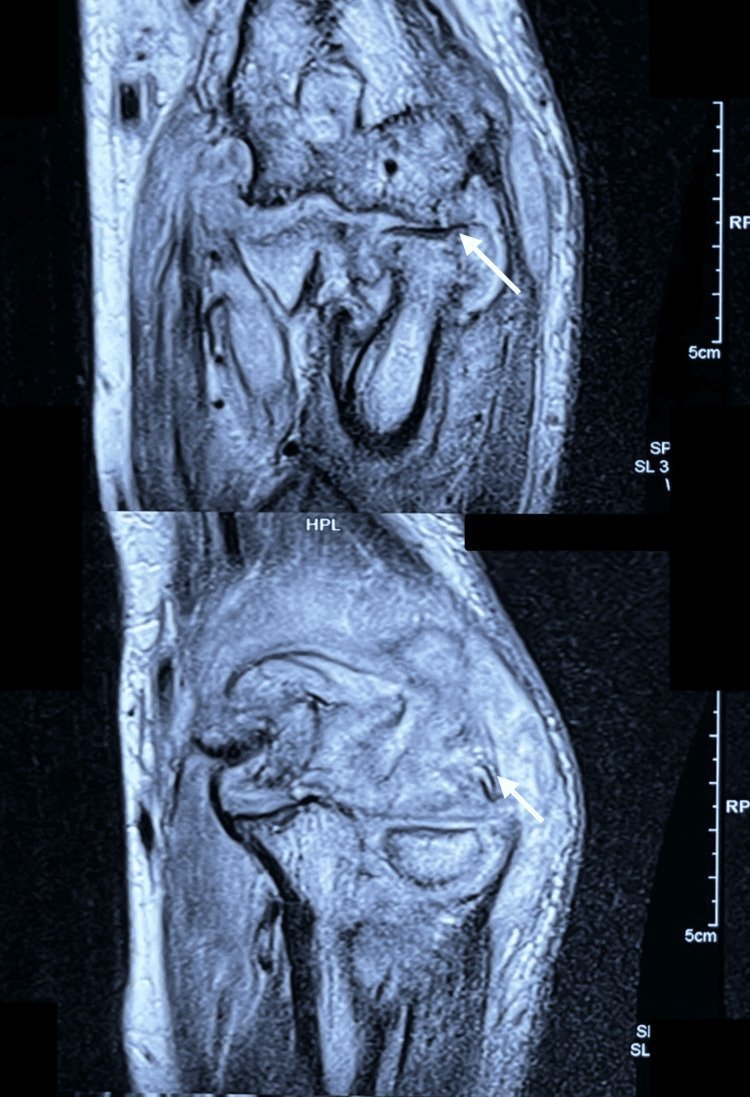
MRI of the elbow demonstrating erosive osteoarticular involvement. MRI demonstrated extensive periarticular soft tissue involvement with erosive changes at the elbow involving the distal humerus and proximal ulna (arrows), associated with synovial thickening and periarticular soft tissue involvement. These findings were consistent with tuberculous arthritis in the appropriate clinical context.

MRI of the left elbow showed extensive periarticular soft tissue involvement with osseous erosions affecting the distal humerus and proximal ulna. The overall imaging appearances were concerning for tuberculous arthritis with associated abscess or phlegmon formation, while septic arthritis remained a differential diagnosis based on imaging alone (Figure 6).

To establish a definitive diagnosis and exclude alternative aetiologies, including malignancy and pyogenic infection, CT-guided fine-needle aspiration of the left elbow lesion was performed. Cytopathological examination demonstrated granulomatous inflammation consistent with TB, confirming the diagnosis in the appropriate clinical and radiological context. In view of multifocal skeletal involvement affecting the spine, sacroiliac joint with psoas extension, and peripheral joint disease of the elbow, a diagnosis of disseminated skeletal TB was made. Alternative causes of granulomatous inflammation, including sarcoidosis, fungal infection, atypical mycobacterial infection, and brucellosis, were considered during the diagnostic evaluation; however, the overall clinical presentation, imaging findings, histopathological results, and subsequent response to anti-tuberculous therapy supported the diagnosis of disseminated skeletal TB.

Treatment

A 12-month anti-tuberculous regimen was advised due to multifocal skeletal involvement: a fixed-dose combination of first-line agents (rifampicin, isoniazid, pyrazinamide, and ethambutol; 4FDC) for the initial two months, followed by continuation therapy with rifampicin and isoniazid (2FDC) for a further 10 months. Non-steroidal anti-inflammatory drugs (NSAIDs) were prescribed for symptomatic pain control for one month, followed by use as needed. The extended treatment duration was selected because of multifocal osteoarticular involvement, including spinal disease, consistent with commonly recommended management strategies for skeletal TB to reduce the risk of relapse [10].

Outcome and follow-up

Two months after initiating anti-tuberculous therapy, the patient demonstrated marked symptomatic and laboratory improvement. Pain severity improved from 8/10 (despite NSAID use) prior to treatment to 2/10 without NSAIDs. Weight increased from 58 kg to 62 kg. Total leukocyte count decreased from 15.87 K/µL to 7.59 K/µL, ESR improved from 74 mm/hour to 28 mm/hour, and CRP decreased from 147.48 mg/L to 7.41 mg/L (Table 1), consistent with an early favourable response to therapy. Pain severity was recorded using a standard 0-10 Numeric Rating Scale (NRS), a validated and freely available clinical assessment tool [12]. No proprietary classification systems, staging criteria, or licensed scoring instruments were used in this report.

**Table 1:** Clinical and inflammatory marker response after two months of anti-tuberculous therapy. Table summarising changes in clinical parameters and inflammatory markers before initiation of anti-tuberculous therapy and after two months of treatment, demonstrating marked clinical and biochemical improvement. Abnormal laboratory values are in bold. Abbreviations: CRP, C-reactive protein; ESR, erythrocyte sedimentation rate; NSAIDs, non-steroidal anti-inflammatory drugs. Pain severity was assessed using the Numeric Rating Scale (0-10) [12].

Parameter	Before anti-tuberculous treatment	After 2 months of treatment	Reference range
Pain (numeric rating scale)	8/10 (with NSAIDs)	2/10 (without NSAIDs)	0-10
Weight	58 kg	62 kg	-
Total leukocyte count	15.87 K/µL	7.59 K/µL	4.0-11.0 K/µL
ESR	74 mm/hour	28 mm/hour	0-15 mm/hour
CRP	147.48 mg/L	7.41 mg/L	<5 mg/L

## Discussion

Disseminated skeletal TB is an uncommon form of extrapulmonary TB and remains a recognised diagnostic challenge because it often presents with non-specific musculoskeletal symptoms and a slow, progressive course that resembles more common inflammatory and pyogenic conditions [1-3]. Although osteoarticular TB represents a small proportion of overall TB disease, delayed recognition is frequent and contributes to preventable complications, including structural joint destruction, deformity, and neurological compromise in spinal disease [2-4]. TB remains highly prevalent in South Asia, including Bangladesh, where extrapulmonary and musculoskeletal manifestations continue to be commonly encountered in clinical practice. In such endemic settings, disseminated skeletal involvement may occur even in the absence of overt pulmonary disease, further complicating early recognition and contributing to diagnostic delay. The present case is clinically important because it demonstrates concurrent involvement of the lumbar spine, sacroiliac joint with psoas extension, and a peripheral joint (elbow), a multifocal pattern that broadens the differential diagnosis and increases the likelihood of delayed or inappropriate initial management. The coexistence of spinal, sacroiliac, and peripheral joint involvement in a single patient is unusual and contributed significantly to the diagnostic complexity in this case.

Diagnostic challenge and differential diagnosis

In routine acute medical and rheumatology practice, the combination of axial pain, sacroiliitis, and systemic symptoms may prompt consideration of seronegative spondyloarthropathy, reactive arthritis, and other inflammatory arthritides. However, fever, weight loss, and rapid functional decline should prompt reconsideration and prioritisation of infection, particularly in TB-endemic settings and in patients with metabolic comorbidities such as diabetes mellitus [1,5]. Similarly, progressive monoarthritis of a large joint accompanied by fever is commonly managed as septic arthritis until proven otherwise. In such cases, early empirical antibiotics are appropriate; however, tuberculous arthritis should remain in the differential diagnosis when symptoms evolve over weeks, radiographs demonstrate erosive changes, and imaging suggests periarticular soft tissue extension rather than isolated synovitis [2,3]. In this patient, elbow involvement with extensive periarticular soft tissue changes initially raised concern for septic arthritis; however, the chronicity and multifocal distribution were more consistent with TB.

The spinal findings in this case also reflect classic pitfalls. Early spinal TB may mimic degenerative disease or inflammatory back pain, and plain radiographs may be normal or non-specific in the early stages [2,4]. Even when vertebral destruction becomes apparent, differentiating TB from pyogenic spondylodiscitis may be difficult on clinical grounds alone, particularly when fever and raised inflammatory markers are present. Certain radiological patterns, especially paravertebral soft-tissue extension and associated collections, are more suggestive of tuberculous infection compared with typical pyogenic disease [3,6]. In the current case, the coexistence of destructive vertebral changes with paravertebral involvement, sacroiliitis, and a psoas collection strongly increased the likelihood of TB and supported urgent pursuit of tissue diagnosis.

Role of imaging in multifocal skeletal TB

MRI is the preferred modality for suspected spinal and osteoarticular TB because it is sensitive for early marrow involvement, soft tissue extension, and occult abscess formation, all of which are critical for both diagnosis and management planning [6-8]. In sacroiliac TB, MRI can demonstrate joint space involvement, adjacent marrow oedema, and associated collections before abnormalities appear on plain radiographs [6,7]. Similarly, in peripheral joint TB, MRI can better delineate synovial thickening, erosions, and periarticular abscess/phlegmon formation, supporting targeted aspiration or biopsy and improving differentiation from purely inflammatory arthropathy [7,8]. In this patient, MRI was central to identifying the full multifocal extent of disease and provided a clear rationale for targeted tissue confirmation.

Despite the high diagnostic value of MRI, radiology alone cannot establish the diagnosis. Imaging findings may be suggestive but continue to overlap with pyogenic infection, inflammatory arthritis, and neoplastic processes, particularly in atypical locations or when only one site is involved [6-8]. Therefore, imaging should be integrated with clinical context and pursued alongside diagnostic sampling where possible, particularly in multifocal skeletal disease.

Importance of tissue diagnosis in paucibacillary disease

Osteoarticular TB is frequently paucibacillary, and microbiological confirmation may be difficult using smear microscopy alone [2,3,9]. Tissue sampling, therefore, remains the cornerstone of diagnosis, with histopathological evidence of granulomatous inflammation providing strong diagnostic support and enabling appropriate treatment in the correct clinical context [9,10]. Modern diagnostic approaches increasingly rely on combined histology with microbiological and molecular testing, as nucleic acid amplification tests can improve diagnostic yield and shorten time to confirmation compared with culture alone [9,11]. In this case, CT-guided aspiration from the elbow lesion confirmed granulomatous inflammation consistent with TB, allowing definitive diagnosis and preventing prolonged diagnostic uncertainty. This step is especially important in disseminated skeletal TB, where misclassification as inflammatory spondyloarthropathy may lead to inappropriate immunosuppression and accelerated disease progression. This case also highlights the importance of obtaining tissue diagnosis when imaging findings are suggestive but not definitive.

Treatment considerations and clinical response

The standard approach to musculoskeletal TB relies on multi-drug anti-tuberculous therapy, with regimen selection and treatment duration guided by disease site, severity, and clinical response [9-11]. In this patient, treatment was initiated with an intensive phase of four first-line agents, followed by continuation therapy, with a planned total duration of 12 months due to spinal and multifocal skeletal involvement. Early improvement in symptoms and inflammatory markers supported a favourable response to therapy, including resolution of fever, substantial reduction in pain and functional limitation, weight gain, and marked improvement in leukocytosis and inflammatory indices. This underscores the importance of timely diagnosis and treatment in preventing progression to irreversible structural damage and disability in skeletal TB [2-4].

Clinical implications and learning value

This case reinforces several practical messages. First, clinicians should consider TB early in patients presenting with subacute musculoskeletal symptoms, especially when multiple sites are involved or when systemic features are present [1-3]. Second, sacroiliac and peripheral joint involvement can coexist with spinal disease and may mimic inflammatory rheumatological conditions; therefore, diagnostic anchoring should be avoided when constitutional symptoms or imaging features raise suspicion for infection [3,6]. Third, MRI should be used proactively to define disease extent and to identify collections that may require targeted sampling or drainage [6-8]. Finally, tissue diagnosis should be pursued promptly in suspected musculoskeletal TB to confirm diagnosis and avoid inappropriate treatment pathways [9-11].

In summary, disseminated skeletal TB may present with concurrent axial and peripheral joint involvement and can closely resemble septic arthritis or inflammatory spondyloarthropathy. Early recognition, appropriate cross-sectional imaging, and timely tissue diagnosis are essential to guide definitive treatment and reduce the risk of long-term morbidity.

## Conclusions

Disseminated skeletal TB is an uncommon but clinically significant manifestation of extrapulmonary TB that may present with concurrent axial and peripheral joint disease, closely mimicking septic arthritis and inflammatory spondyloarthropathies. This case underscores the importance of maintaining a high index of suspicion for TB in patients with subacute progressive musculoskeletal symptoms and systemic features, particularly in endemic settings and when multiple skeletal sites are involved. Early cross-sectional imaging to define the extent of disease and timely tissue diagnosis are essential to establish the diagnosis, guide appropriate anti-tuberculous therapy, and prevent irreversible structural damage and long-term functional impairment.
